# Characterization and mesoscale modeling of an enhanced UHMWPE fabric treated with *bis*-diazirine: multicriteria crosslinker selection and surrogate-based inverse parameter estimation

**DOI:** 10.1007/s42114-025-01367-1

**Published:** 2025-07-01

**Authors:** Mahshid Mahbod, Stefania F. Musolino, Jeremy E. Wulff, Reza Vaziri, Abbas S. Milani

**Affiliations:** 1 Composites Research Network, The University of British Columbia, Vancouver, B.C. Canada; 2 Department of Chemistry, University of Victoria, Victoria, B.C. Canada; 3 Materials and Manufacturing Research Institute, School of Engineering, The University of British Columbia, Kelowna, B.C. Canada

**Keywords:** Crosslinked UHMWPE fabric, Mechanical properties enhancement, Experimental characterization, Mesoscale modeling, Surrogate-based inverse optimization

## Abstract

Ultra-high molecular weight polyethylene (UHMWPE) woven fabrics are commonly used in armor applications due to their superior biaxial mechanical and physical properties. In this study, three different diazirine-based crosslinker options were initially considered as the chemical treatment applied to a dry UHMWPE plain weave to improve a range of its properties. The optimum crosslinker was then selected using a VICOR multicriteria decision-making model. Specifically, through the bias-extension and yarn pull-out tests, it was observed that the optimum crosslinker significantly enhanced (> 100%) the crossover interactions between the warp/weft yarns. Subsequently, a mesoscale finite element model was developed to predict both the tensile and shear responses of the untreated and treated fabrics. In developing this model, an inverse analysis was employed to capture the effect of yarn transverse elastic modulus and the friction at the crossovers—two properties that are known to be difficult to measure directly in the weave form of yarns. These parameters were sampled using a design of computational experiments and then optimized via a surrogate-based model. Finally, challenges presented by the crosslinking at the single yarn level during characterization are discussed and resolved numerically. For both the treated and untreated fabrics, the mesoscale model is shown to predict the material behavior accurately.

## Introduction

Woven fabrics have been increasingly employed in various engineering applications due to their high specific strength, stiffness, and impact resistance, among other desired properties [1]. In fabricating such bidirectional fibrous structures, ultra-high molecular weight polyethene (UHMWPE) fibers have become popular choice of fibers in armor applications owing to their high toughness, high tensile, and shear strengths, along with low wear friction and low volumetric mass density [2, 3]. In addition to the base UHMWPE, its various functional composites have been proposed using material modifiers to enhance the fabric performance. For example, studies have been performed on the analysis of adding shear thickening fluids which can possibly improve the impact energy absorption of the UHMWPE fabric [4–7]. In another attempt, Musolino et al. [8] impregnated a UHMWPE weave with diazirine-based crosslinkers. Their experimental results indicated that the tear resistance and tensile strength properties increased in the modified fabric by 54% and 35%, respectively.

Woven fabrics are composed of weft/warp yarns that are intertwined, each including thousands of fibers [9]. Considering such fibrous architecture, different micro/meso/macro approaches have been applied to model their mechanical response under basic deformation modes [10–12]. The macro-scale is the component geometry (full fabric) scale, where the fabric is considered a fully homogenized material. At this level, the geometrical and material parameters such as sheet bending stiffness and fiber volume fraction are included, while the mesoscale geometrical characteristics such as yarn cross-section and weave shape of yarns are ignored (or implicitly accounted for) [13]. The mesoscale is the scale of homogenized yarns, which may be modeled as a continuum shell or solid element. The main goal of performing this level of analysis is to investigate the fabric’s internal structure and weaving principle [10]. It should be noted that the macro-scale mechanical behavior of the fabrics can be significantly affected by their mesoscale level response, depending on the complexity of a given fabric and applied loading mode [14]. The micro-scale model is the scale of fibers. The fibers’ arrangement and interactions within the yarn are considered at this level and can predict the effective (homogenized) yarn properties at the meso-level [15]. Considering a balance of computational speed and accuracy, mesoscale modeling may be considered a very suitable mid-ground approach for understanding basic defamation mechanisms underlying new fabric material systems while avoiding excessive computational costs.

In the past investigations, different mesoscale models have been developed to predict the mechanical behavior of woven fabrics subject to base tensile and shear loading modes. The accuracy of such models is largely affected by the quantity of data and details used in the model to describe the geometry and the fiber/yarn material properties for each given weave. In most such meso-level studies, a unit cell geometry is developed based on Peirce’s model [16], where the yarn path is defined by a sinusoidal function [17], and the yarn cross-section is modeled by rectangular [18], lenticular [19], or ellipsoidal shape [20]. In addition, for automating the fabric meso-level geometry creation, the TexGen software has been widely applied due to its ability to automatically generate yarn paths and different cross-section shapes [21–24]. Lin et al. [25] developed a mesoscale fabric simulation model using TexGen, where a linear-elastic transversely isotropic material behavior was applied to fibrous yarns. Their results demonstrated that the model has a very good accuracy against experimental results, which has been also supported by similar studies under tensile and shear response of fabrics [26–29]. In such a modeling approach, the longitudinal elastic modulus of yarns is often obtained by performing the single yarn tensile testing and using an inverse identification (fitting). However, the *transverse elastic constants of yarn and the friction coefficient between the warp/weft yarns* (at crossovers) in the fabric are challenging parameters to measure directly, and their values in numerical models are often set based on assumptions or trial-and-error [30–32]. As a more systematic approach, in the study by Komeili and Milani [13], the yarn transverse elastic modulus was determined through a conventional inverse method by achieving a minimal error between numerical and experimental data. In that study, however, the finite element (FE) model itself was used as the data generation tool during optimization search, thus making the inverse identification process costly.

It is worth adding that inverse optimization methods have also been employed successfully in other composite modeling applications. Zeman and Sejnoha [33] investigated the effect of imperfections in defining unit cells of fabrics. Namely, they incorporated the morphology of imperfect material systems using a set of statistical descriptors, which were then considered optimization parameters under an inverse method to generate desired representative unit cells. In another work, Milani and Nemes [34] employed an inverse method to characterize the mechanical behavior of thermoplastic composite materials, including the influence of noise factors on the mechanical response. Umbharatwala et al. [35] conducted an inverse optimization study to determine the optimal mesh size for a mesoscale fabric model under ballistic impact while maintaining modeling accuracy. To address computational time during inverse optimizations, however, especially when dealing with micro/meso FE models, none of the previous studies considered the “surrogate”-based/meta modeling approach.

In the present study, we aim to characterize a new UHMWPE functionalized composite fabric, treated by a set of diazirine crosslinker options [8], and accordingly model its mechanical behavior at the mesoscale using FE while adapting a surrogate-based inverse parameter identification framework to capture the unknown yarn properties. To this end, first, a comprehensive experimental analysis is conducted to compare different UHMWPE fabric groups, impregnated with different types of bis-diazirine crosslinkers, under tensile, bias-extension, Peirce’s bending, tear, and puncture tests. Next, in order to select the optimal crosslinker type, a formal multi-criteria VIKOR decision-making method is employed, while allowing different weights to be assigned by the designer/decision maker to the objective functions based on the end-application design requirements. Ultimately, the best-performing crosslinker is selected and used for the subsequent mesoscale simulations. For the simulations, the geometrical model of the plain weave fabric unit cell has been developed using micro-CT scanning. For data sampling, as part of the surrogate model development, a design of (computer) experiments approach has been employed considering both the yarn transverse elastic modulus and the crossover (warp/weft) coefficient of friction properties. As stated earlier, these two properties are known to be difficult to measure directly in the as-given crimp form of the fabrics, and hence, the presented surrogate-based identification framework would provide a new means to estimate these parameters numerically.

### Remark

*Surrogate-based optimization* is known as a technique that uses a metamodel (e.g., a response surface) to find the global minimum of an objective function that would be otherwise expensive or time-consuming to evaluate directly. The method is particularly suited for objective functions that are the result of complex/time-consuming meso/micro level simulations.

In summary, the main contribution of the present work lies in integrating a formal multi-criteria decision-making (MCDM) approach along with surrogate-based inverse parameter estimation towards experimental optimization and numerical modeling of a novel *bis*-diazirine-based crosslinked UHMWPE fabric.

## Experimental

### Materials

**Fig. 1:**
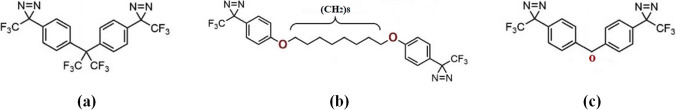
Different bis-diazirine crosslinker options referred to as **a** Gen I, **b** Gen IIIA, and **c** Gen IIIC

The base untreated (control) fabric was a 200 denier UHMWPE plain weave with an areal density of 75 g/m^2^. The fabric samples were treated with chemically compatible *bis*-diazirine crosslinker options identified in an earlier work of the authors [8]. Three different crosslinkers selected are referred to hereafter as Gen I, Gen IIIA, and Gen IIIC (Fig. 1). Gen IIIA crosslinker (Fig. 1b) is an electron-rich bis-diazirine, featuring an 8-carbon aliphatic chain that connects two aryl diazirine units via ether linkages. This crosslinker features a flexible chain connecting two aryl diazirine units, which theoretically enhances its ability to bridge the host polymer strands more effectively than the shorter chains found in Gen I and Gen IIIC crosslinkers. Moreover, the tether’s molecular weight was intentionally selected to minimize the risk of explosion, which is a concern for densely packed compounds of Gen IIIC, where the energetic diazirine groups are inadequately offset by the mass distributed in other parts of the molecule.

**Fig. 2:**
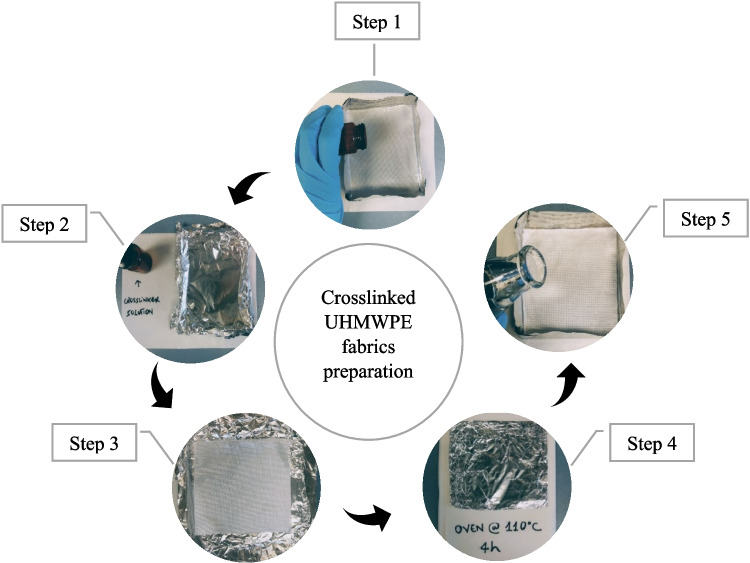
Visualization of the fabric within each step (listed in Sect. 2.1) during the preparation of crosslinked samples

The steps of preparing the crosslinked fabric samples are visually summarized in Fig. 2, and outlined below.

- **Step 1:** The fabric was impregnated with the desired molecule by placing the sample into a close-fitting aluminum pan filled with a crosslinker solution in pentane at the appropriate concentration. This concentration was calculated to impregnate the fabric with 1 wt% of crosslinker, but in order to compensate for crosslinker deposited on the sides and bottom of the aluminum pan, an extra 0.25 wt% was added: for a given piece of fabric, the amount of crosslinker in the solution was 1.25 wt% of its mass.
- **Step 2:** The bath was covered with aluminum foil and left to sit at room temperature for 30 min.
- **Step 3:** The cover was removed to allow the pentane to evaporate in a well-ventilated fume hood for 20 min.
- **Step 4:** After evaporation, the impregnated fabric sheets were wrapped in aluminum foil and placed in an oven at 110 °C for 4 h. If not covered by aluminum, the crosslinker could evaporate (loss of the impregnated mass) before reacting at a high temperature, most likely due to increased surface exposure.
- **Step 5:** After thermal activation of the crosslinker, the samples were weighed to determine the total mass of reacted crosslinker with fabric. Each piece was washed three times for 5 min at room temperature with pentane to remove unreacted crosslinkers and possible side products that were not attached to the fabric. After drying the treated fabrics, each sample was weighed again to determine the mass of reaction products lost during solvent washing.

To better understand the effect of the crosslinker, two groups of untreated and vehicle control material samples were fabricated, and the results of respective models were compared accordingly. Finally, a similar fabrication procedure used for the crosslinked (treated) samples was employed to prepare the so-called vehicle control samples; i.e., following all steps of Fig. 2 but without adding a crosslinker in the pentane bath. The untreated (actual control) samples refer to the as-received woven 200 denier UHMWPE fabric without any further chemical processing. As such, a difference in response between the control and vehicle control samples would help isolate the effects of other treatment steps, such as oven heating.

### Mechanical characterization of the treated fabrics

**Fig. 3:**
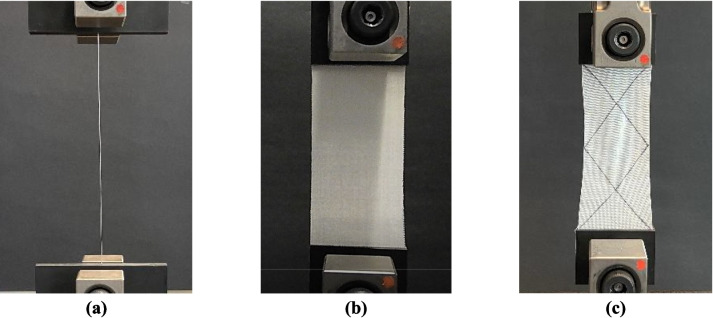
Experimental setup for **a** the tensile test of single yarns, **b** the tensile test of fabrics, and **c** the bias-extension test

The tensile (both at single yarn and fabric levels), bias-extension (shear), Peirce’s bending, puncture, yarn pull out, and tear tests were conducted to characterize and compare the effects of different crosslinkers on the base fabric properties. For the tensile and shear tests, an Instron 5969 dual column load frame was employed, with a quasi-static loading rate of 5 mm/min. The sample dimensions were 75 mm × 250 mm (following ASTM D5035). Each end of the sample was inserted 50 mm into the grips, resulting in a gauge length of 150 mm between the grips. In the bias-extension test, under the same loading rate, the samples were aligned in such a way that the warp and weft yarns were oriented at ± 45° with respect to the global loading direction. Three samples of each group of fabrics were tested under all tests to assess consistency in data collection. Moreover, the tensile test on the single yarn of the optimum material group was performed to provide the input data for the subsequent mesoscale model. This test was conducted considering 250 mm of gage length according to ASTM D2265/D2265M, with a loading rate of 5 mm/min. The screw side-action grips were used, and sandpapers were utilized to prevent the yarns’ slippage. The experimental setups for the single yarn tensile test, fabric tensile test, and bias-extension test are shown in Fig. 3.

**Fig. 4:**
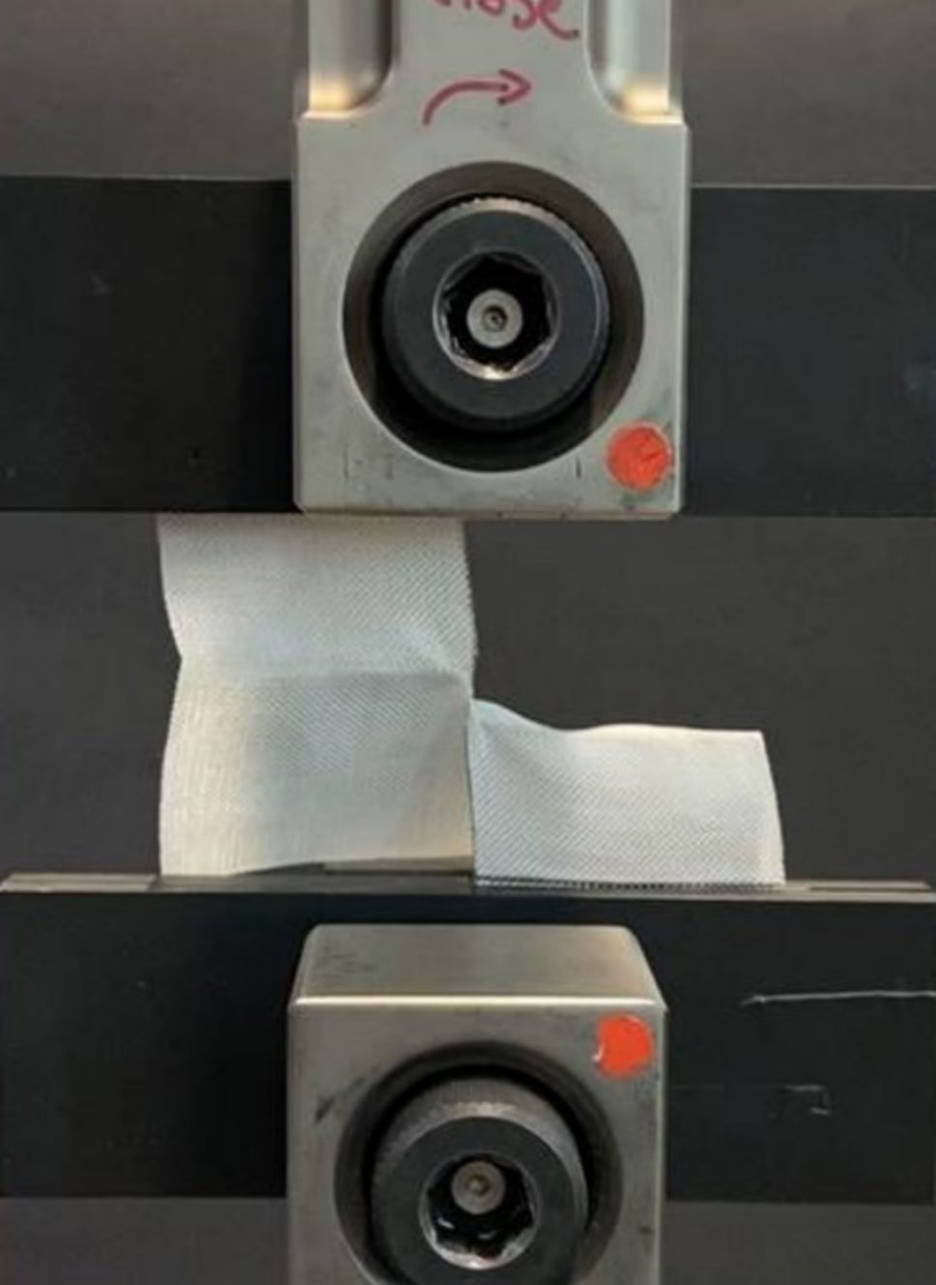
The tear test sample and fixture

**Fig. 5:**
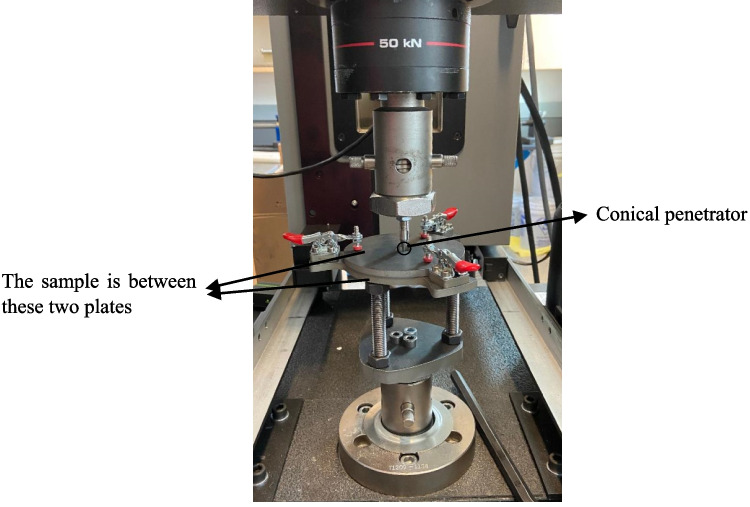
The puncture test setup

Next, the tear resistance of the fabrics was characterized according to ASTM D2261. A 100 mm × 100 mm sample was prepared with a centered edge cut of 50 mm in length—introduced using a hot wire cutter. The two “legs” of the sample were then clamped symmetrically, with 25 mm clamped on each side, in the universal testing system (Instron, Series 5969) shown in Fig. 4. The sample was pulled apart at a rate of 30 mm/min while the force-extension curve was recorded. The puncture test was performed using the same dual column load frame where the puncture fixture was designed in-house and attached to the jigs of the machine (Fig. 5). Samples of size 70 mm × 70 mm and a conical penetrator were used, and according to ASTM F1342/F1342M, the loading rate was set at 508 mm/min [36]. It is worth noting that in the study [37], it was shown that the UHMWPE fabric exhibits rate-dependent behavior only at much elevated rates (e.g., under drop tower at 3 m/s).

**Fig. 6:**
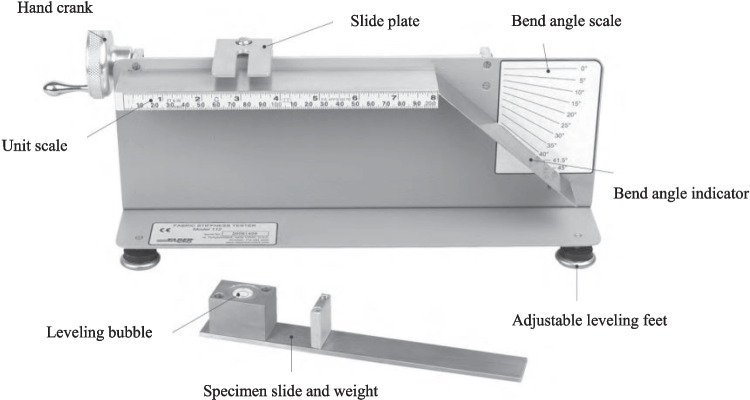
Peirce bending test setup used for fabric flexural stiffness characterization

In order to characterize the out-of-plane bending behavior of the fabrics, the Peirce bending test [38] was employed. The specimen of size 25 mm × 200 mm was placed on the horizontal platform with the length of the specimen parallel to the platform edge. Then, the clamped specimen was moved gradually manually until the edge of the sample touched the 41.5° indicator (Fig. 6). The bending length of the specimen was then read, and the flexural rigidity was determined as follows [38]:

1 $$G=9.81 \times {10}^{-12} \times w \times {c}^{3}$$

Assuming that the bending length, *c*, has units of mm and the areal weight, *w*, is expressed in g/m^2^, then the flexural rigidity, *G*, will have the unit of N·m.

**Fig. 7:**
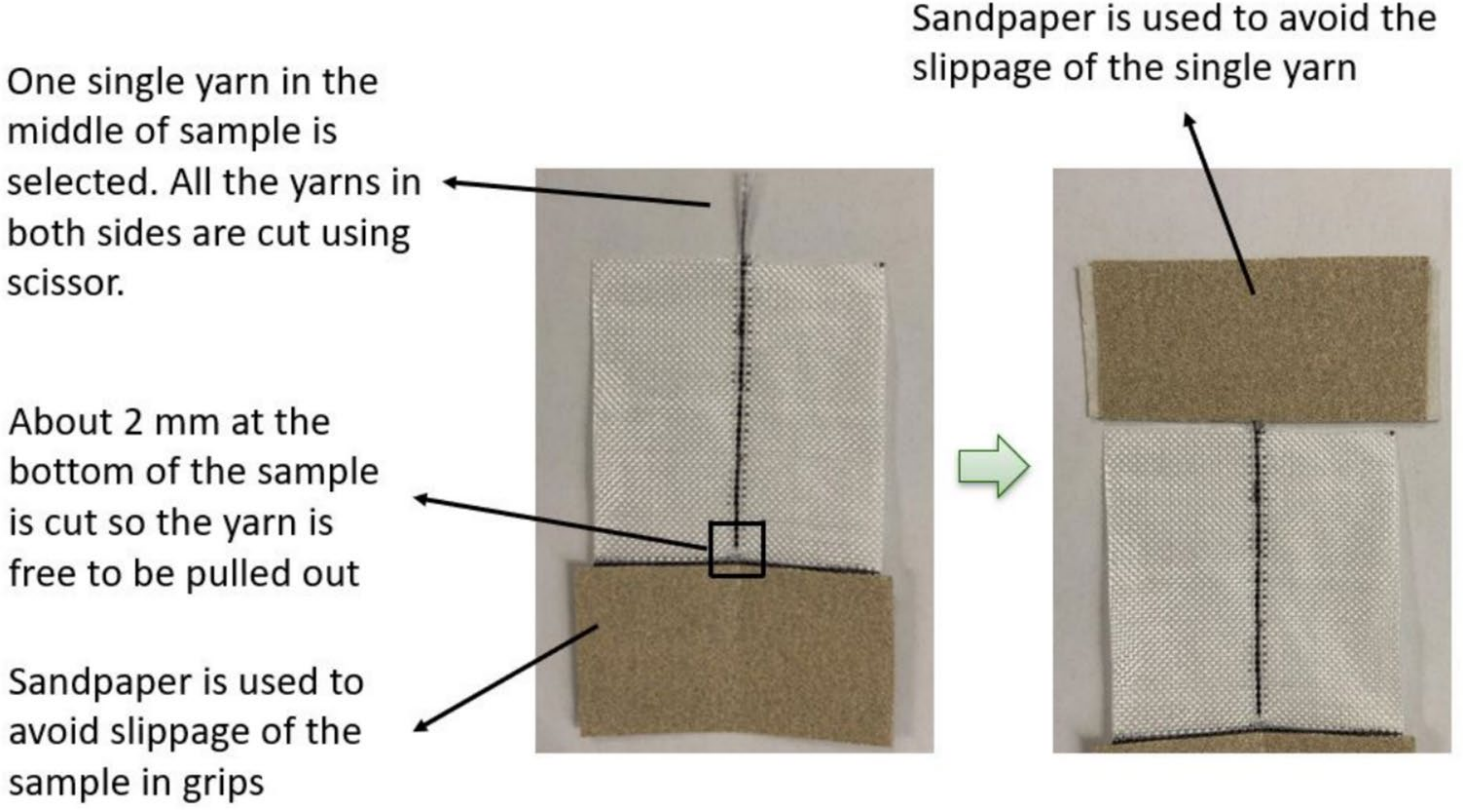
A yarn pull-out sample employed during characterization to characterize crossover interactions

Finally, the yarn pull-out test was performed in order to directly investigate the interaction between the warp and weft yarns and the effect of crosslinking on this interaction. The lower part of the sample of 50 mm × 100 mm was clamped between the grips. Moreover, the middle yarn is cut at the bottom and displacement was applied to this yarn by the upper grip (Fig. 7). Five samples of each material group were tested. There is no specific standard for the loading rate used in yarn pull-out tests; however, based on a review of previous studies, we selected a rate of 10 mm/min, as it was equal to or below the rates commonly used and remains low enough to ensure quasi-static conditions [39–41].

## Mesoscale finite element model

### Geometry and mesh generation

**Fig. 8:**
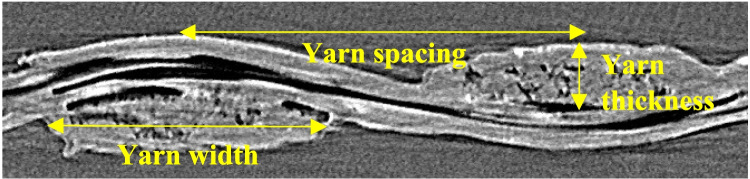
Cross-section of the scanned image of the untreated UHMWPE fabric

The first step in FE modeling of the fabric samples was to characterize their mesoscale structures. To do so, microcomputed tomography (micro-CT) was conducted (using ZEISS Xradia MicroXCT-400) to create virtual 3D images of the base fabric specimen. This method is known as a non-destructive imaging technique that provides information on the internal morphological structure of an object without destroying it [42]. Figure 8 shows the cross-section of the scanned image of an untreated fabric. The thickness and width of the yarns were determined from four different cross-sections, and the average of these dimensions was calculated. Specifications and the measured geometrical properties of the material are provided in Table 1. In the next step, the CAD model of the single unit cell of the material, as well as its meshing, was generated using TexGen. A three-dimensional tetrahedral mesh with an element size of 0.037 mm was applied to the model, resulting in a total of 7360 elements for a single unit cell. The model was created using solid elements. A sinusoidal shape was assigned to the warp and weft yarns [17], as also evident in Fig. 8.

**Table 1:** Geometrical specification of the base (untreated) UHMWPE fabric

Geometrical specifications	Value
Nominal yarn thickness, warp, µm	129
Nominal yarn thickness, weft, µm	119
Nominal yarn spacing, warp, µm	740
Nominal yarn spacing, weft, µm	685
Nominal yarn width, warp, µm	617
Nominal yarn width, weft, µm	585

### Loading and boundary conditions

**Fig. 9:**
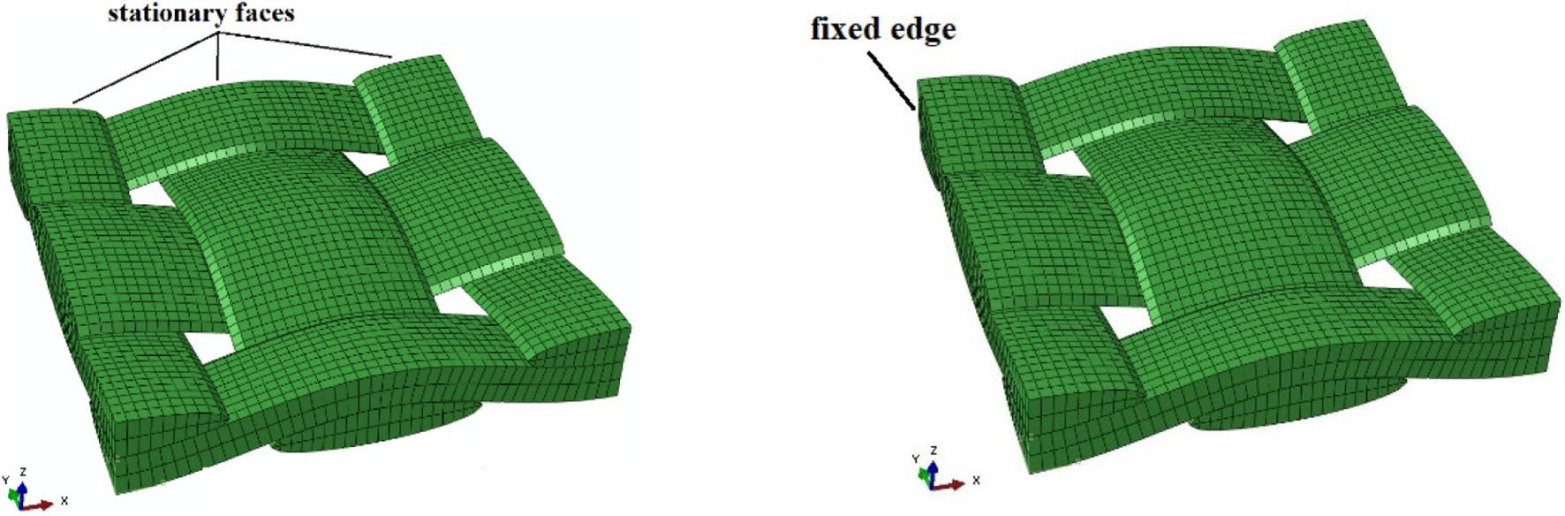
The boundary and loading conditions of the unit-cell model subjected to **a** tensile loading and **b** shear loading

The loading conditions were defined based on each test scenario (tensile and shear loadings). Figure 9 shows the displacement boundary conditions applied to the model under each loading scenario. As demonstrated in Fig. 9a, for tensile loading, to avoid rigid body motion of elements, one of the free sides of the unit cell is constrained in the direction normal to loading. In shear (picture frame) loading mode, on the other hand, one corner/edge is fixed, and the displacement is applied to the opposite corner. In addition, the nodes of each edge have the same degrees of freedom [23, 43]. In order for the single unit cell to represent the mechanical behavior of the whole material, a periodic boundary condition [23, 43–45] is applied to the unit cell. To ensure that the unit cell accurately represents the behavior of the fabric ply, the periodic boundary conditions were applied such that the elements on the boundary surfaces remain tied to their respective surfaces during deformation. As a result, the nodes along the edges of the unit cell are not independent but are constrained to move together in a way that maintain surface continuity, i.e., mimicking the behavior observed in a single layer of fabric (e.g., refer to [45] for mathematical representation).

### Yarn material properties

A transversely isotropic material model was used to simulate the mechanical behavior of the fabric yarns. According to physical observations, Poisson’s ratio of yearns is nearly zero in the “dry” form [30–32, 46] (a very small value can be assigned in the FE model to avoid numerical instabilities). Moreover, in this case, assuming the fiber direction in the yarn to be 1, the transverse shear modulus, ${G}_{23}$, is defined as follows:

2 $${G}_{23}=\frac{{E}_{2}}{2\left(1+{\vartheta }_{23}\right)}$$

The shear moduli in all three directions are considered to be equal:

3 $${G}_{23}={G}_{12}={G}_{13}$$

Moreover:

4 $${E}_{2}={E}_{3}$$

The longitudinal elastic modulus of the untreated fabric was obtained through the single yarn tensile testing discussed in Sect. 2.2. However, the other material parameters of the yarns could not be obtained through physical tests accurately (given the soft nature of the fabric in dry form), as also has been reported in earlier studies [30–32]. Similarly, efforts were made to experimentally determine the friction coefficient between yarns at crossovers within the fabric in the weave form, but the results were not promising given the highly cohesive nature of the crosslinker. Consequently, in the following sections, a design of experiments methodology along with a surrogate-based inverse (bi-objective) optimization routine is proposed to determine these model parameters (details to follow in Sects. 4.2 and 4.3).

## Results and discussion

### Crosslinker selection and characterization

Recalling Sect. 2, three different crosslinked fabric groups were evaluated, subjected to various loading conditions. As presented in the Appendix, a formal MCDM analysis was employed to select the optimum crosslinker alternative for the current UHMWPE fabric. Based on the results of the MCDM, the Gen IIIA demonstrated a superior overall effect on improving the mechanical performance of the fabric compared to Gen I and Gen IIIC. Accordingly, in the following sections, we will focus on this optimum crosslinker and discuss the main experimental tests required for the subsequent FE model calibration and validation.

#### Tensile properties

Tensile tests were performed on (Gen IIIA-) treated, control, and vehicle control, as well as their respective single yarns. The fabric-level stress–strain curve was also used to validate the subsequent mesoscale models, while the tensile test results of the single yarns were fed directly as the input to the mesoscale simulations. The elastic modulus and maximum stress of the crosslinked, untreated (control), and vehicle control fabrics and yarns are compared in Table 2.

**Table 2:** Comparison between the tensile properties of different yarns and fabric material groups

Material level	Fabric			Single yarn		
Property	Vehicle control	Untreated	Crosslinked	Vehicle control	Untreated	Crosslinked
Elastic modulus (GPa)	5.76 ± 0.11	6.18 ± 0.16	6.23 ± 0.08	10.12 ± 0.49	20.01 ± 0.78	N/A
Tensile strength (MPa)	186.68 ± 35.83	201.97 ± 33.36	251.58 ± 28.12	94.01 ± 19.13	368.18 ± 66.05	N/A

Table 2 shows that the tensile strength is considerably higher in the crosslinked UHMWPE fabric compared to the untreated (control) and vehicle control samples. Moreover, a slight increase in the elastic modulus was observed in the crosslinked fabric compared to the untreated and vehicle control samples. In contrast with these fabric-level tensile test results, at the single yarn level, based on Table 2, the crosslinked samples’ tensile testing was deemed unrealistic (noted as “N/A”). The initial observation was that a single yarn in the crosslinked state could not be manually separated cleanly from the crosslinked fabric (due to the highly cohesive nature of the crosslinker over the entire fabric). Consequently, an attempt was made to apply the crosslinking process on the fabric and single yarns separately, but then the ensuing yarn could not be fully representative of the mechanical behavior of the same fabric sample.

Nevertheless, it is interesting that, regardless of the application of the diazirine crosslinker itself, the process used for crosslinking the material (Sect. 2) has affected the mechanical performance of yarns much more significantly than the fabric-level properties. Namely, the comparison of the vehicle control and untreated samples in Table 2 shows this effect, while in both fabric-level and yarn-level tests, the vehicle control has lower properties compared to the control. As mentioned earlier, the vehicle control samples were prepared in the same way as the crosslinked samples but without adding the crosslinker. However, per Table 2, the crosslinked sample is found to be stronger than both other sample groups. This, in turn, confirms that the chemical reaction made through the application of the crosslinker onto the fabric is a dominant step in the entire process.

##### Hypothesis

To explain the above tensile test results, it is hypothesized that the crosslinker significantly affects the yarns “interaction” at the crossovers, which should then affect the shear response as well as the yarn pull-out force of the fabric considerably. To prove this hypothesis, the bias-extension and yarn pull-out tests were employed next (where the response of both is known to be closely driven by the crossover friction property [38–40]).

#### Bias-extension (shear)

**Fig. 10:**
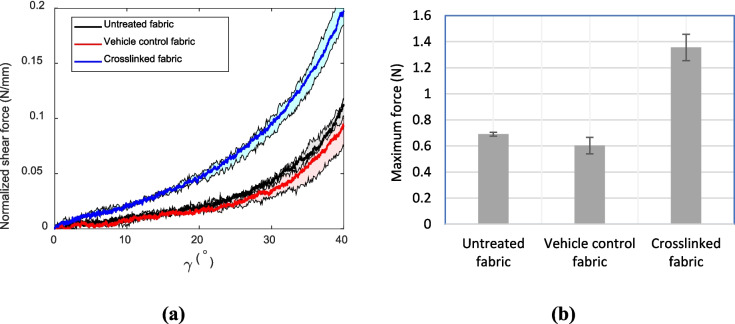
The normalized shear force-shear angle curve results of the fabrics in the bias-extension test (shaded areas represent standard deviations). **b** The maximum load response to pull-out of the single yarn in different fabric groups

The normalized shear force-shear angle curves for the three fabric groups are shown in Fig. 10a, proving the above-stated hypothesis where the crosslinked fabric shows a stiffer response under the shear loading. The reason for a lower response in the vehicle control compared to the control in both bias-extension and tensile tests can be attributed to the softening (or possible decomposition) of the yarns due to the heat/oven application step (110 °C for 4 h) in the process (Sect. 2).

#### Yarn pull-out

Figure 10b presents the maximum force values observed in the yarn pull-out tests. This figure clearly demonstrates that the maximum force increases in the crosslinked samples (by over 100%) compared to the vehicle control and untreated samples. Again, similar to the earlier tests, the vehicle control shows a lower response compared to the control.

### Modeling of the untreated fabric, integrated with surrogate-based inverse optimization using genetic algorithm

Recalling Sect. 3.2, a transversely isotropic material model was used to mimic the mechanical behavior of the fabric yarns. Although the longitudinal elastic modulus of the untreated fabric was achieved by performing a single yarn tensile test (Table 2), the transverse elastic modulus, as well as friction coefficient at crossovers (in the weave form), could not be estimated through direct experimental tests. Similar experimental infeasibilities were reported in [31, 47], while these two parameters theoretically affect the mechanical response of the model subject to the tensile and shear loadings. To address this, we employed a surrogate-based multi-objective parameter identification framework to inversely estimate these parameters. Namely, a response surface model was developed whereby the above material parameters could be optimized by minimizing the error between the numerical and experimental data subjected to both tensile and shear loadings concurrently. To do so, ${E}_{2}$ and the friction coefficient (*f*) were considered optimization/search variables. For sampling, a full factorial design by considering three levels of each variable was proposed (Table 3). The numerical simulations subjected to tensile and shear loadings were performed for each of the nine designs. The errors between numerical and experimental results according to both loadings are shown in Table 3. The error in tensile loading is defined by the different elastic moduli measured numerically and experimentally. However, the error in the shear loading was determined by calculating the difference between the numerical and experimental results of *normalized* shear force (following [42]) at ten equally distanced shear angles.

**Table 3:** Full-factorial design tested to identify the effect of unknown model parameters of the intreated fabric and to develop the response surface models

Design (treatment)	${E}_{2} (\text{MPa})$	*f*	Error in tensile loading	Error in shear loading
1	0.07	0.06	− 4.26	− 38.07
2	0.07	0.09	− 4.00	− 31.65
3	0.07	0.12	− 3.74	− 27.14
4	0.10	0.06	0.90	− 14.13
5	0.10	0.09	1.16	− 7.80
6	0.10	0.12	1.42	− 2.60
7	0.13	0.06	4.00	+6.10
8	0.13	0.09	4.52	+12.76
9	0.13	0.12	4.77	+20.33

Based on the error values in Table 3, two regression models under tensile and shear loadings were identified:

5 $$Error\_tensile\;loading=36.85-704E_2-22.6f+3394E_2^2-50f^2+358.3E_2f$$

6 $$Error\_shear\;loading=15-1988E_2+1921f+9909E_2^2-10577f^2+1725E_2f$$

**Fig. 11:**
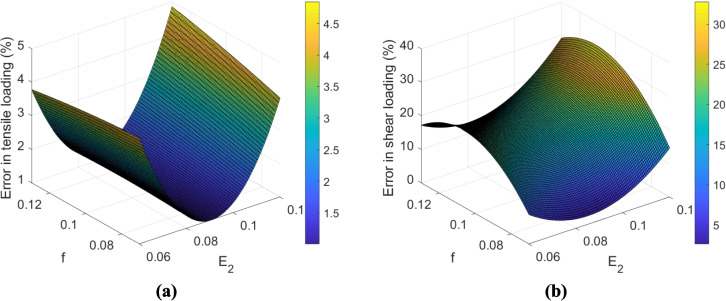
The surface model indicating the relationship between **a** error in tensile loading and **b** error in shear loading with the friction coefficient and transverse elastic modulus in the untreated fabric

To provide a visual understanding of these error function models, they are plotted in Fig. 11. As indicated in Sect. 1, the mechanical behavior of the material subjected to tensile loading is not only dependent on the yarn elastic modulus linearly, given the curvature of the response surface. This can also be comprehended from Eq. (4). In this equation, the coefficients of ${E}_{2}$ and ${{E}_{2}}^{2}$ are significantly higher than the coefficients of $f$ and ${f}^{2}$. As illustrated in Fig. 11a, the error in tensile loading is approximately constant at different friction coefficients. Consequently, the effect of friction coefficient is negligible in this mode, and the terms related to $f$ and ${f}^{2}$ could be removed in Eq. (4). Similarly, the error in shear loading is plotted as a function of the transverse elastic modulus and friction coefficient. It is observed that the shear behavior of the material is non-linearly dependent on ${E}_{2}$ and $f$.

In the next step, the genetic algorithm (GA) in MATLAB was utilized to optimize the values of these unknown model parameters. The optimization was bi-objective, based on a weighted sum of the two error functions. The weights assigned to the two (sub)objective functions in each subjective design are tabulated in Table 4. In this table, the first and last rows represent the single objective optimization by considering the objective function as the error in tensile and shear loading only, respectively. In the second row, equal weights were assigned to both objective functions to achieve a model that could well simulate the mechanical behavior of the material for general-purpose in-plane loadings.

**Table 4:** Assigned weights to the aggregated objective function to optimize the mechanical response of the untreated UHMWPE fabric, subjected to tensile and shear loadings

Weighting cases	Assigned weight to the first objective function (tensile loading)	Assigned weight to the second objective function (shear loading)
1	1	0
2	0.5	0.5
3	0	1

Based on these different weighting combinations, the mechanical properties of the UHMWPE yarn were derived, as shown in Table 5. It should be noted that, as demonstrated in Table 5, the minimum in both Fig. 11 a and b occurs at approximately the same point. In other words, both objective functions result in nearly the same design. Consequently, in the performed bi-objective optimization, assigning different weights to the objective functions did not have a significant effect on the results in the present case study. In all cases, the optimum friction coefficient is identified to be 0.06, while there is a slight variation in the value of transverse elastic modulus.

**Table 5:** The mechanical properties of the untreated UHMWPE yarn identified using multi-objective GA

Weighting case	${E}_{2}={E}_{3} (\text{MPa})$	*f*	Error in tensile loading (%)	Error in shear loading (%)
1	100	0.060	1.00	2.82
2	96.4	0.060	1.06	2.60
3	95.2	0.060	1.10	2.58

**Fig. 12:**
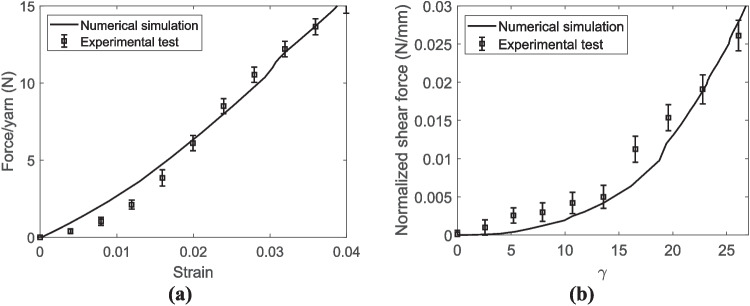
The comparison between numerical and experimental results for the untreated UHMWPE fabric subjected to **a** tensile loading and **b** shear loading

The low coefficient of friction of UHMWPE fabrics has been reported as a drawback of this material in the literature [48, 49]. The present study also resulted in a relatively low coefficient of friction between yarns (Table 5). Eventually, preferring equal weighting between the two deformation modes, the optimum transverse elastic modulus was set at 964 MPa based, and the friction coefficient at 0.060. The comparison between the ensuing numerical and experimental results in terms of force/yarn versus strain curves under tensile loading and normalized shear force versus shear angle subjected to shear loadings is represented in Fig. 12. As observed in these figures, there is a fairly good agreement between numerical and experimental results.

### Modeling of the crosslinked fabric, integrated with surrogate-based inverse optimization using genetic algorithm

Similar to Sect. 4.2, the material parameters of the crosslinked UHMWPE fabric were obtained, but this time, the longitudinal elastic modulus also needed to be considered the third search variable (also refer to “N/A” in Table 2). Consequently, a Taguchi L9 design [50] was proposed by considering three tree-level parameters of longitudinal elastic modulus, transverse elastic modulus, and the friction coefficient (reducing the number of trials from 27 to 9, per Table 6). Moreover, the errors in tensile and shear loadings for each design are indicated in the same table.

**Table 6:** The Taguchi L9 design for the crosslinked UHMWPE fabrics

Designs (treatments)	${E}_{1} (\text{GPa})$	${E}_{2} (\text{MPa})$	*f*	Error in tensile loading	Error in shear loading
1	14	70	0.06	− 31.49	− 41.76
2	14	100	0.09	− 23.01	− 17.15
3	14	130	0.12	− 17.35	+8.67
4	20	70	0.09	− 13.17	− 33.83
5	20	100	0.12	− 1.00	− 5.72
6	20	130	0.06	− 6.64	+2.70
7	26	70	0.12	− 16.82	− 26.55
8	26	100	0.06	+16.82	− 45.84
9	26	130	0.09	+28.70	+15.16

Next, based on the obtained errors in Table 6, two regression models under tensile and shear loadings were defined:

7 $$\begin{array}{ll}Error\_tensile\;loading=266.7-\\19.99E_1-1835E_2+834.8f+\\0.43E_1^2+0.62E_2^2-4615f^2\\+27.26E_1E_2-1.61E_1f\end{array}$$

8 $$\begin{array}{ll}Error\_shear\;loading=214.19-\\8.54E_1-2484E_2+862.7f+\\0.33E_1^2+6146E_2^2+2815f^2+\\30.07E_1E_2-77.67E_1f\end{array}$$

**Fig. 13:**
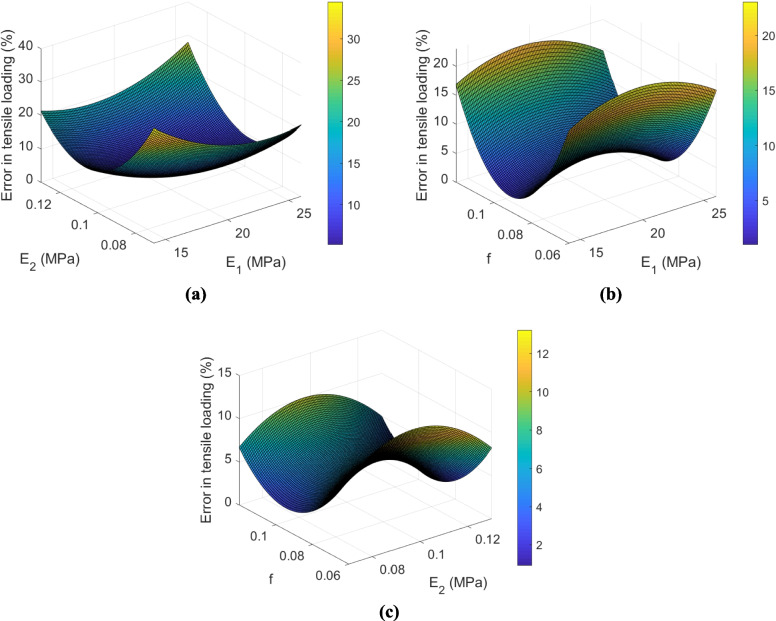
The surface contours in tensile loading mode of the crosslinked fabric

**Fig. 14:**
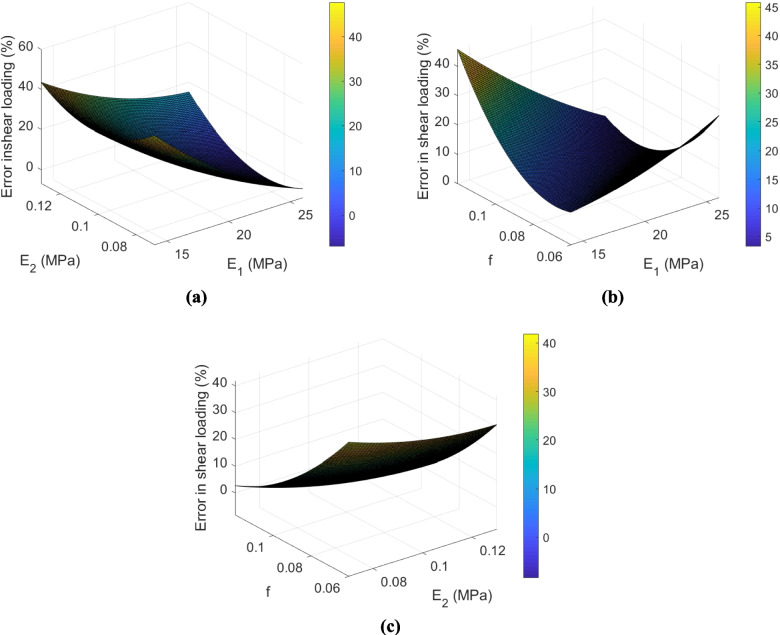
The surface contours in shear loading mode of the crosslinked fabric

To better visualize these regression surface models, they are plotted in Figs. 13 and 14. Similar to the untreated UHMWPE fabric in the earlier section, in the crosslinked UHMWPE fabric, the optimized variables in the linear-elastic transversely isotropic yarn model were determined based on the above surrogate models and GA (Table 7). The assigned weights to the objective functions in each trial are also tabulated in Table 4. As indicated in this table, the friction coefficient in the crosslinked fabric is considerably higher (~ 100% higher) than that in the untreated fabric (compare Tables 5 and 6), which is in good agreement with the results of yarn pull out test, and also the hypothesis made earlier in Sect. 4.1.1. As demonstrated in Fig. 10, the interaction between yarns was increased in the crosslinked UHMWPE fabric compared to the untreated fabric.

**Table 7:** The inversely identified mechanical properties of the crosslinked UHMWPE yarn using multi-objective GA

Weighting case	${E}_{1} (\text{GPa})$	${E}_{2}={E}_{3} \left(\text{MPa}\right)$	*f*	Error of 1 st objective function (%)	Error of 2nd objective function (%)
1	20.25	105	0.12	0.89	2.21
2	21.82	107	0.12	2.15	0.32
3	20.23	111	0.12	1.20	1.05

**Fig. 15:**
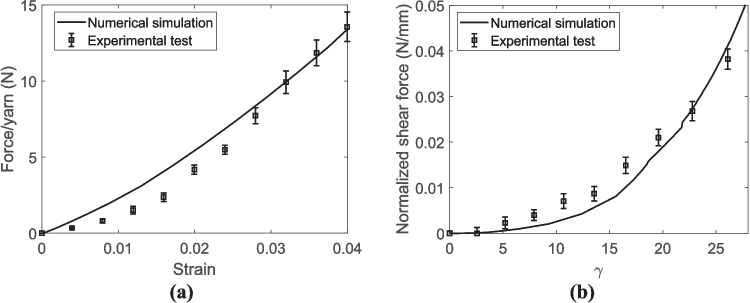
The comparison between numerical and experimental results of the crosslinked UHMWPE fabric subjected to **a** tensile loading and **b** shear loading

The comparison between numerical and experimental results in terms of force per yarn versus strain curves under tensile loading and normalized shear force versus shear angle subject to shear loadings is represented in Fig. 15. The results indicate that the numerical model could successfully predict the mechanical behavior of the crosslinked UHMWPE fabric under both loadings. It was also convincing to notice that, considering equal weights for the two objective functions per Table 7, the estimated longitudinal modulus of the crosslinked yarn is 21.82 GPa, which is larger than those directly measured for the control and vehicle control samples in Table 2.

## Conclusions

In this study, a comprehensive experimental analysis using the tensile (at both single yarn and fabric levels), bias-extension (shear), Peirce’s bending, puncture, yarn pull-out, and tear tests (similar to, e.g., [51–54]) was conducted to characterize and compare Bis-diazirine-based crosslinked UHMWPE fabrics. A multi-criteria decision-making (MCDM) approach was employed to identify the optimal crosslinker among the provided options. It was shown that the selection of the top-ranked alternative depends closely on the fabric’s intended application and the specific criteria required by a designer. In most application scenarios exemplified, Gen IIIA crosslinker ranked the highest using the VIKOR method. Next, focusing on this crosslinker, the meso-level modeling part of the study aimed at the ability to predict the mechanical response of the Gen IIIA crosslinked-UHMWPE fabric subjected to tensile and shear loading conditions. A linear-elastic transversely isotropic material model was utilized to define the mechanical properties of the yarns. An L-9 Taguchi design of experiments and a subsequent surrogate-based bi-objective inverse optimization were performed to identify the unknown elastic constants as well as the crossover friction coefficient in the model. The error between response surface predictions and experimental results in tensile and shear loadings was considered the objective functions during optimization. The chemically processed crosslinkers applied to the UHMWPE fabric resulted in the improvement of its mechanical properties both under tensile (by 25%) and shear (by 65%) modes of loading. The main underlying property for these increases was deemed to be the higher interactions between weft and warp yarns at the crossovers due to crosslinking—a hypothesis which was tested via the bias-extension and yarn pull-out tests. To further investigate this factor, inverse identification results revealed that the coefficient of friction at yarn crossovers was 0.06 for the untreated fabric and 0.12 for the crosslinked fabric, representing a 100% increase.

The comparison between numerical and experimental results validated the accuracy of the developed mesoscale model. The model may serve as a foundational framework for future design studies, particularly for simulating more complex loading scenarios such as ballistic impact of the material system. While mesoscale simulations are prohibitively time-consuming for such complex scenarios, necessitating the utilization of macro-scale simulations, the latter inherently lack the capability to incorporate mesoscale material parameters such as yarn width and spacing. In a future work, a hybrid approach integrating both simulation scales could be explored.

## Figures and Tables

**Fig. 16:**
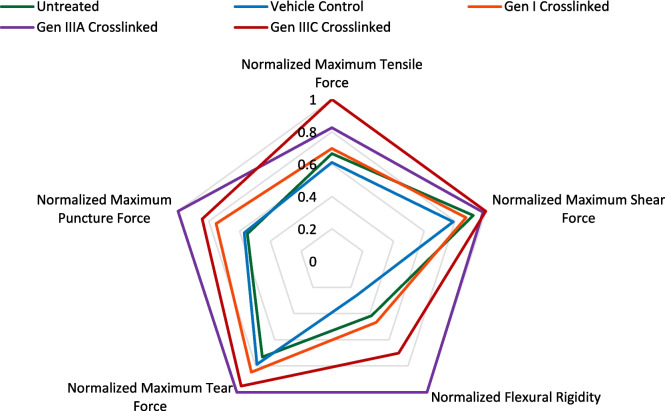
Spider diagram comparing material groups

## Data Availability

Datasets that were generated or analyzed are included as part of the main text.

## Appendix. Selection of the optimum crosslinker using MCDM

Table 8 presents the test results for maximum tensile force, maximum shear force, flexural rigidity, maximum tear force, and maximum puncture force across all the sample groups. The latter mechanical performance metrics were subjectively chosen as a demonstrative example of general decision criteria for the selection of UHMWPE fabric, which can be refined based on different end-use applications (more discussion to follow below during criteria weighting). For visual comparison, a spider diagram illustrating the performance of the five material groups under these metrics is shown in Fig. 16. In this figure, the objective functions are normalized by dividing each group’s values by the maximum observed across all sample groups. As shown, the Gen IIIA crosslinked fabric outperformed all other groups in terms of flexural rigidity, puncture resistance, and tear resistance. Although the Gen IIIC fabric exhibited better performance in terms of maximum tensile force compared to Gen IIIA, the difference between these two groups in terms of maximum shear force is not significant. Notably, both Gen IIIA and Gen IIIC fabrics demonstrated agreeable overall performance compared to the Gen I crosslinked, untreated, and vehicle control samples in all tests.

**Table 8:** Comparison between different fabric material groups under different mechanical performance criteria

Decision criteria	Untreated (control)	Vehicle control	Gen I crosslinked	Gen IIIA crosslinked	Gen IIIC crosslinked
Maximum tensile force (N)	3045	2796	3190	3778	4578
Maximum shear force (N)	938	805	890	1003	1021
Flexural rigidity (μjoule/m)	0.67	0.42	0.75	1.61	1.13
Maximum tear force (N)	783	843	907	1071	1021
Maximum puncture force (N)	9.65	10.01	13.22	17.57	14.81

To make a more informed/systematic decision, the VIKOR decision-making method [50] was employed to score the material groups. The VIKOR method is known to be particularly effective for ranking and selecting among alternatives that involve conflicting (design) criteria with different units. This is achieved through a compromise ranking and evaluating the proximity of each alternative to an imaginary ideal solution [55]. The steps of this optimization method are as follows:

o **Step 1:** The values of the decision matrix (here Table 8) are normalized. The various alternatives are denoted as ${x}_{1}, {x}_{2},\dots , {x}_{5}$. For an alternative ${x}_{i}$, the merit of the *j*^th^ aspect is denoted by ${f}_{ij}$; i.e., ${f}_{ij}$ is the value of *j*th criterion function for the alternative ${x}_{i}$. *m* is the number of alternatives; *n* is the number of criteria.
o **Step 2:** Determination of the best and the worst values for each of the criteria functions: The best ${f}_{j}^{*}$ and the worst ${f}_{j}^{-}$ values are defined as follows.
  A-1 $${f}_{j}^{*}={\text{max}}_{i}{f}_{ij}, {f}_{j}^{-}={\text{min}}_{i}{f}_{ij}; i=\text{1,2},\dots ,m; j=\text{1,2},\dots ,n$$

if the *j*^th^ function is to be maximized (benefit-type); and

A-2 $${f}_{j}^{*}={\text{min}}_{i}{f}_{ij}, {f}_{j}^{-}={\text{max}}_{i}{f}_{ij}; i=\text{1,2},\dots ,m; j=\text{1,2},\dots ,n$$

if the *j*^th^ function is to be minimized (cost-type).

o **Step 3:** Computing the values ${S}_{i}$ and ${R}_{i}$ for each alternative by the relationships:
  A-3 $${S}_{i}=\sum_{j=1}^{n}\frac{{w}_{j}\left({f}_{j}^{*}-{f}_{ij}\right)}{\left({f}_{j}^{*}-{f}_{j}^{-}\right)}; i=\text{1,2},\dots ,m; j=\text{1,2},\dots ,n$$
  A-4 $${R}_{i}={\text{max}}_{j}\left[\frac{{w}_{j}\left({f}_{j}^{*}-{f}_{ij}\right)}{\left({f}_{j}^{*}-{f}_{j}^{-}\right)}\right]; i=\text{1,2},\dots ,m; j=\text{1,2},\dots ,n$$
o **Step 4:** Computing the values ${Q}_{i}$ by:
  A-5 $${Q}_{i}=v \frac{\left({S}_{i}-{S}^{*}\right)}{\left({S}^{-}-{S}^{*}\right)}+\left(1-v\right)\frac{\left({R}_{i}-{R}^{*}\right)}{\left({R}^{-}-{R}^{*}\right)}; i=\text{1,2},\dots ,m$$
  where ${S}^{*}= {\text{min}}_{i}{S}_{i}$, ${S}^{-}= {\text{max}}_{i}{S}_{i}$, ${R}^{*}= {\text{min}}_{i}{R}_{i}$, and ${R}^{-}= {\text{max}}_{i}{R}_{i}$; and $v$ is a weight for the strategy of the “majority of criteria”, which is assumed to be 0.5 here.
o **Step 5:** Ranking the alternatives by ${Q}_{i}$. The less the value of ${Q}_{i}$, the better the ranking of the respective alternative.

To apply this MCDM method in the current study, it was also necessary to subjectively determine the weights assigned to each design criterion in Table 8. The selection of appropriate weights depends on the material’s intended application. Let us as a 1 st scenario assume a general application for the UMWPE fabric where a novice designer may assign equal weighting to all five criteria. The results of the VIKOR under this scenario are shown in Table 9. As indicated in the table, the Gen IIIA crosslinked fabric achieved the highest ranking in this case, followed by the Gen IIIC crosslinked option.

**Table 9:** The results of VICOR method in different criteria weighting scenarios

Alternatives (material groups)	Ranking in 1 st scenario	Ranking in 2nd scenario	Ranking in 3rd scenario	Ranking in 4th scenario
Untreated (control)	4	5	5	5
Vehicle control	5	4	4	4
Gen I crosslinked	3	3	3	2
Gen IIIA crosslinked	1	1	1	3
Gen IIIC crosslinked	2	2	2	1

As a 2nd scenario, we considered an alternative specific application owing to the high performance of the UHMWPE fabrics: a body armor with a stabbing resistance requirement. In this case, the tear resistance and puncture resistance become the most important design criteria. Therefore, we assigned weights of 0.33 to each of these two criteria, and 0.11 to the remaining three. The results of the VIKOR method under this case are presented in Table 9, where the rankings for the top three alternatives remain unchanged, with the only difference being that the untreated fabric now ranks fifth, while vehicle control ranks fourth.

Another important consideration for wearable armor materials would be flexibility/comfort. As a result, a high flexural rigidity is not always desirable; instead, a lower flexural rigidity may be preferable for particular application cases. Although the flexural rigidity of the fabric in this study was not high enough to hinder its use as wearable, we opted to test a 3rd scenario where the material is preferred to bend as easily as possible. In this scenario, flexural rigidity was treated as a cost function in the VIKOR method, meaning it should be now minimized. The results for this case are shown in Table 9, where the rankings remain the same as the previous scenario, indicating the dominance effect of main design criteria including the tear and puncture resistance.

Finally, let us consider a hypothetical application for casual clothing using the same fabric. In this case, the threat of puncture (stabbing resistance) would be less critical, but the focus shifts to having a material that is very flexible and exhibits a high maximum tear force. For this 4th scenario, we assigned equal weights of 0.33 to both maximum tear force and flexural rigidity (which is treated as a cost function similar to the preceding scenario), with weights of 0.11 assigned to all other criteria. The results of the VIKOR method for this case in Table 9 show that the Gen IIIC crosslinked fabric ranks first, followed by the Gen I crosslinked fabric as the second choice.

Overall, per Table 9, the selection of the best crosslinker material option is highly dependent on its intended application. However, in most cases, the Gen IIIA demonstrated a superior effect compared to the other alternatives.
